# Visual Detection of SARS-CoV-2 RNA by Conventional PCR-Induced Generation of DNAzyme Sensor

**DOI:** 10.3389/fmolb.2020.586254

**Published:** 2020-12-23

**Authors:** Anbalagan Anantharaj, Soon Jyoti Das, Patil Sharanabasava, Rakesh Lodha, Sushil K. Kabra, Tarun Kumar Sharma, Guruprasad R. Medigeshi

**Affiliations:** ^1^National Capital Region - Biotech Science Cluster, Translational Health Science and Technology Institute (THSTI), Faridabad, India; ^2^Department of Pediatrics, All India Institute of Medical Sciences, New Delhi, India

**Keywords:** colorimetric assay, COVID-19, SARS-CoV-2, DNAzyme, sensor, real-time-PCR

## Abstract

The gold standard for the diagnosis of SARS-CoV-2, the causative agent of COVID-19, is real-time polymerase chain reaction (PCR), which is labor-intensive, expensive, and not widely available in resource-poor settings. Therefore, it is imperative to develop novel, accurate, affordable, and easily accessible assays/sensors to diagnose and isolate COVID-19 cases. To address this unmet need, we utilized the catalytic potential of peroxidase-like DNAzyme and developed a simple visual detection assay for SARS-CoV-2 RNA using a conventional thermal cycler by the PCR-induced generation of DNAzyme sensor. The performance of RT-PCR DNAzyme-based sensor was comparable to that of real-time PCR. The pilot scale validation of RT-PCR DNAzyme-based sensor has shown ~100% sensitivity and specificity in clinical specimens (nasopharyngeal swab, *n* = 34), with a good correlation (Spearman *r* = 0.799) with the Ct-value of fluorescence probe-based real-time PCR. These findings clearly indicate the potential of this inexpensive, sensitive, and specific molecular diagnostic test to extend our testing capabilities for the detection of SARS-CoV-2 to curtail COVID-19 transmission.

## Introduction

The ongoing pandemic of Coronavirus Disease 2019 (COVID-19) caused by severe acute respiratory syndrome coronavirus-2 (SARS-CoV-2) has affected more than nine million people, with close to 500,000 deaths, globally (www.worldometers/.info/coronavirus) (Carter et al., 2020; Mahapatra and Chandra, 2020). Worldwide efforts are on to develop vaccines or therapeutics against SARS-CoV-2, which is a major challenge considering the ongoing pandemic situation. However, rapid screening and early detection of cases in the population may help in containing the spread of the disease. The current diagnostic modalities for SARS-CoV-2 detection primarily fall into two major approaches—nucleic acid amplification-based assay and serodiagnostics (Udugama et al., 2020). The most frequently used approach is the molecular assay that provides confirmatory diagnosis by detecting SARS-CoV-2 viral RNA using reverse transcription polymerase chain reaction (RT-PCR) in a real-time PCR machine. This method demands a sophisticated machine and a highly skilled manpower, and many of these molecular assays also utilize expensive fluorophore-labeled probes (Tang et al., 2020). Access to real-time PCR machine is restricted to few select locations in low-and-middle-income countries (LMICs). Under these circumstances, it is imperative to develop an alternative molecular assay that can be used in conventional thermal cyclers which are inexpensive and easily accessible in many resource-poor settings.

A DNAzyme with peroxidase activity is a well-known DNA enzyme and has been shown to adopt G-quadruplex structures. Hemin, a porphyrin, can non-covalently bind to the G-quadruplex which enhances the peroxidase activity of hemin (Kosman and Juskowiak, 2011; Ghahremani Nasab et al., 2017). This property of hemin conjugated to DNA quadruplex has been used as a strategy in screening ligands of G-quadruplex for a variety of applications which range from single-nucleotide polymorphisms to proteins and nucleic acids and also in drug discovery and diagnostics employing colorimetric or visual detection strategies and also as electrochemical sensors (Cheglakov et al., 2006; Cheng et al., 2009; Won et al., 2011; Bhadra et al., 2014; Peng et al., 2018). We followed a previous strategy of tagging the 5′ end of the primers with sequences complementary to the G-quadruplex which forms a G-quadruplex only upon amplification of the PCR product (Bhadra et al., 2014). A similar strategy has been employed for the detection of viruses such as hepatitis B virus and to detect the HIV-1 gag gene by other studies previously (Yang et al., 2017; Kim et al., 2018). We employed this approach to detect SARS-CoV-2 RNA by conventional PCR. Our assay utilizes the PCR-induced generation of a DNAzyme sensor that acquires a functional G-quadruplex structure in the presence of hemin and KCl and catalyzes the oxidation of 2,2′-azino-bis(3-ethylbenzothiazoline-6-sulfonate) (ABTS) that leads to a greenish-colored product that can be visualized by the naked eye. We believe that this assay may have a major impact on the early and affordable detection of SARS-CoV-2 and limiting the spread of COVID-19 in low- and middle-income settings.

## Materials and Methods

### Ethics

The study was approved by the ethics committee of THSTI THS 1.8.1/(91) dated 13 April 2020. RNA samples from other respiratory virus infections were used from a study described previously (Kumar et al., 2017), which was a cohort of newborns who were followed for the first year of life, for each episode of acute respiratory infection. Nasopharyngeal aspirates were collected to identify the causative respiratory viruses using multiplex real-time PCR assay. This study was approved by the institutional ethics committees of THSTI, Faridabad [THS/1.8.1(12)], and AIIMS New Delhi (IEC/P-13512012 and RP-O6/2O12).

### Reagents

Hemin, KCl, ABTS, and all the primers used in the current study were procured from Merck Life Science Pvt. Ltd. (Germany). Nuclease-free water was purchased from HiMedia Laboratories, India. The Onestep RT-PCR kit was obtained from Qiagen (USA); the OptEIA 3,3′,5,5′-tetramethylbenzidine (TMB) substrate was procured from BD Biosciences, USA. NucleoSpin Gel and PCR Clean-up kit were procured from MACHEREY-NAGEL, Germany.

### Reverse Transcription and PCR-Induced Generation of DNAzyme Sensor

#### Primers

The “N” gene-specific primers were designed according to the complete SARS-CoV-2 genomic sequence (RefSeq ID NC_045512.2). The forward primer (P1A forward DNAzyme 5′-*ACCCACCCACCCACCCAG*CGTTTGGTGGACCCTCAGAT-3′ corresponding to nucleotide 28320 to 28339) and the reverse primer (P1B reverse DNAzyme 5′-*TCCCTCCCTCCCTCCCAG*TGTAGCACGATTGCAGCATTG-3′, corresponding to nucleotide 28,752–28,732) yield an amplicon of ~430 bp. The primer sequences in italics comprise a reverse complementary sequence of a peroxidase-like DNAzyme. These primers on amplification incorporate a functional DNAzyme sequence (5′-CTGGGTGGGTGGGTGGGT-3′/5′-CTGGGAGGGAGGGAGGGA-3′) in the PCR product that acts like a sensor.

#### In Vitro Transcript for Quantification

The region of N gene starting from 28,287 to 29,230 was amplified using the forward primer 5′-GACCCCAAAATCAGCGAAAT-3′ and the reverse primer 5′-GCGCGACATTCCGAAGAA-3′ and cloned into the pGEM®-T-Easy vector (Promega). This clone was linearized using Sac II enzyme and *in vitro* transcribed using the SP6 RNA polymerase (Promega). The transcript was purified and used as a template for generating the standard curve for the limit of detection (LOD) experiments. For all the reactions except the limit of detection, 0.01 ng *in vitro*-transcribed (IVT) RNA was used as a template.

#### Reverse Transcription and DNAzyme-Based Visual Detection of SARS-CoV-2 RNA

RNA (10 μl) isolated from COVID-19-positive or -negative nasopharyngeal/oropharyngeal (NP/OP) swab samples was used in the study. Reverse transcription was performed using Onestep RT-PCR kit (Qiagen) as per the manufacturer's instructions (reaction volume, 25 μl). Reactions were incubated in a thermal cycler (ABI Veriti) for 30 min at 50°C, followed by 95°C for 15 min, 35 cycles of 94°C for 30 s, 50°C for 1 min, and 72°C for 1 min, and a final extension at 72°C for 10 min. The PCR reaction was first cleaned up to remove buffer components and unused primers using a PCR clean-up kit. The amplicon was eluted with 50 μl nuclease-free water. The amplicon was denatured at 92°C for 5 min, followed by the addition of 5 μM hemin and 10 mM KCl, and incubated at room temperature on a rotaspin at 12 rpm for 20 min. Finally, 40 μl TMB/ABTS was added, and color development was observed with naked eyes within 5 min. The intensity of the color was quantified in terms of absorbance at 650/410 nm using SpectraMax M2E Plate reader (Molecular Devices). In parallel, amplicon was resolved using Qiagen QIAxcel capillary electrophoresis system to visualize the amplicon size and intensity.

#### Limit of Detection

The LOD of this assay was established by using a range of IVT RNA (corresponding to 10^1^–10^7^ copies) as a template, followed by monitoring of DNAzyme reaction (as mentioned in section Reverse Transcription and PCR-Induced Generation of DNAzyme Sensor).

#### Assessment of Cross-Reactivity

To ascertain the selectivity of the assay, the stored RNA samples isolated from patients positive for other respiratory viruses, namely, parainfluenza virus (*n* = 1), influenza A virus (*n* = 2), human adenovirus (*n* = 1), rhinovirus (*n* = 4), human coronavirus OC43 (*n* = 1), and human respiratory syncytial virus (*n* = 1) were tested (Kumar et al., 2017).

#### Clinical Evaluation

NP/OP swabs (*n* = 34) were collected in viral transport medium (HiMedia Laboratories India) from individuals suspected to have COVID-19. The RNA was extracted using NucleoSpin RNA isolation kit (MACHEREY-NAGEL), and DNAzyme assay was conducted as described in sections Reagents and Reverse Transcription and PCR-Induced Generation of DNAzyme Sensor. To compare the performance of the developed assay with the current gold standard, i.e., RT-PCR, these samples were tested in parallel on a real-time PCR platform using a diagnostic kit (Altona Diagnostics, Germany). A total of 20 samples were found negative by real-time PCR, and 14 samples were positive. Finally, the diagnostic sensitivity and the specificity of both RT-PCR and DNAzyme-based assay were determined using receiver operating characteristic (ROC) curve using GraphPad Prism 5.0. In addition to this, the performance of both tests was compared in terms of positive prediction value (PPV) and negative predication value (NPV) using MEDCALC (https://www.medcalc.org/calc/diagnostictest.php).

### Statistical Analysis

Statistical tests, as indicated in the figures, were performed using GraphPad Prism software (Version 7.0e). Error bars indicate mean with SEM. All experiments were performed with two replicates at least twice, except the sensitivity determination which was performed only once due to the limited amount of stored RNA. *P* values were estimated by Mann-Whitney test.

## Results

### PCR-Induced Generation of DNAzyme

**Figure 1:**
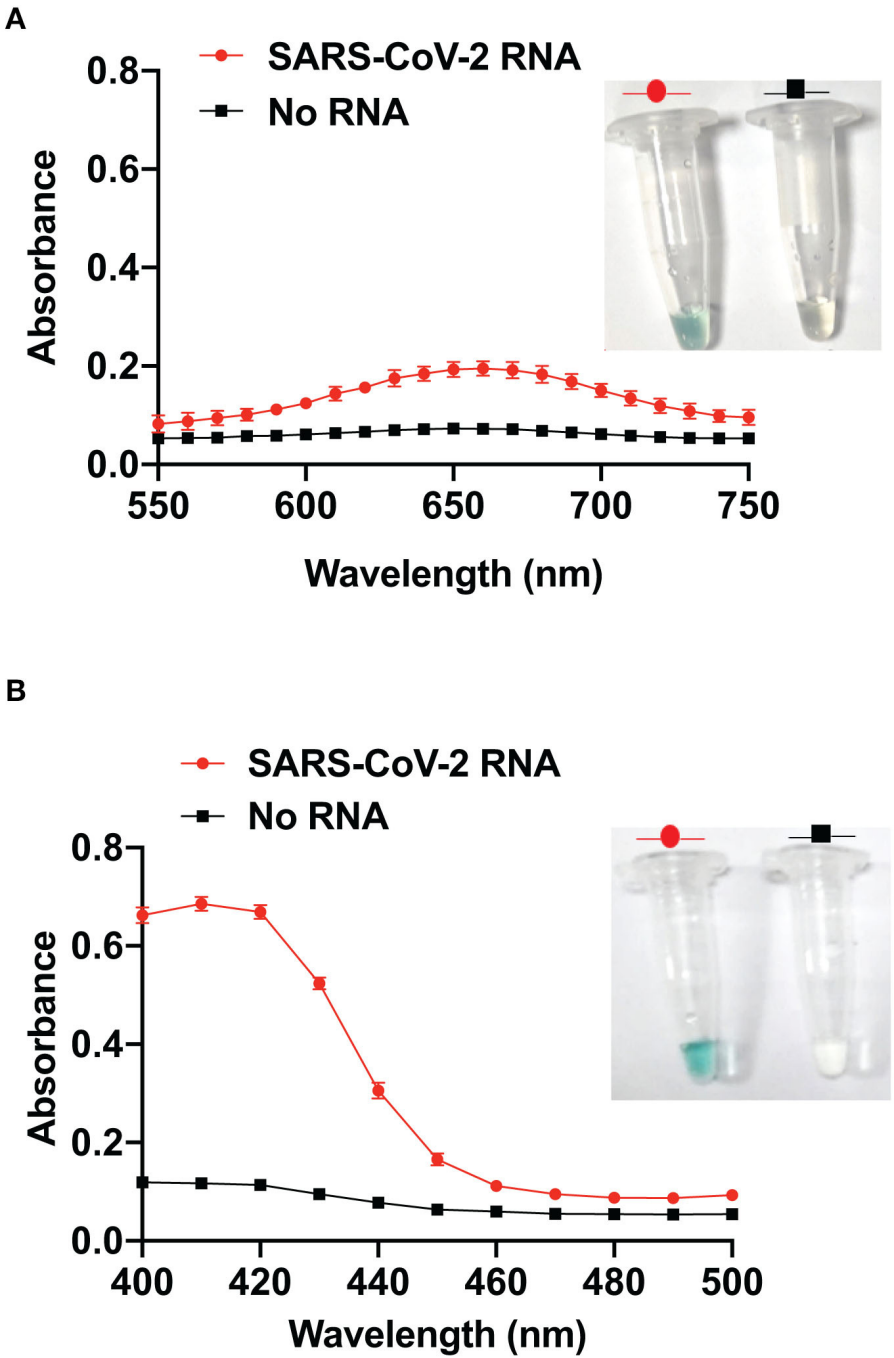
Visual detection of SARS-CoV-2 RNA using DNAzyme sensor. **(A)** Visual detection and its corresponding absorbance spectrum in the presence of TMB. **(B)** Visual detection and its corresponding absorbance spectrum in the presence of ABTS.

Detection of SARS-CoV-2 RNA was based on the principle of PCR-induced generation of functional DNAzyme (Cheglakov et al., 2006; Liu et al., 2018). The P1A forward DNAzyme and P1B reverse DNAzyme primers targeting the “N” gene of SARS-CoV-2 were used in a one-step RT-PCR reaction using a conventional thermal cycler. The primers were designed to allow the PCR-induced formation of functional DNAzyme sequence in the amplicon. After denaturation followed by the addition of KCl and hemin, a G-quadruplex structure is formed within the amplicon, with an ability to catalyze the oxidation of colorless TMB or ABTS to a blue- or green-colored product, respectively. This oxidation or TMB/ABTS yields a visual color change in the presence of viral RNA (Figure 1). The PCR product was also run on QIAxcel capillary electrophoresis platform (Supplementary Figure 1) for further confirmation. It is evident from the absorbance spectrum of the DNAzyme-TMB/DNAzyme-ABTS sensor that the oxidation of TMB/ABTS (a characteristic peak at 650/410 nm) takes place only in the presence of SARS-CoV-2 RNA (Figures 1A,B). No such peak was observed in the negative control reactions where no viral RNA was added (no template control). Interestingly, the ABTS (Figure 1B) produced a stronger signal (absorbance) than the TMB (Figure 1A). Therefore, for all the subsequent experiments, ABTS was used as the substrate.

### Time Kinetics

**Figure 2:**
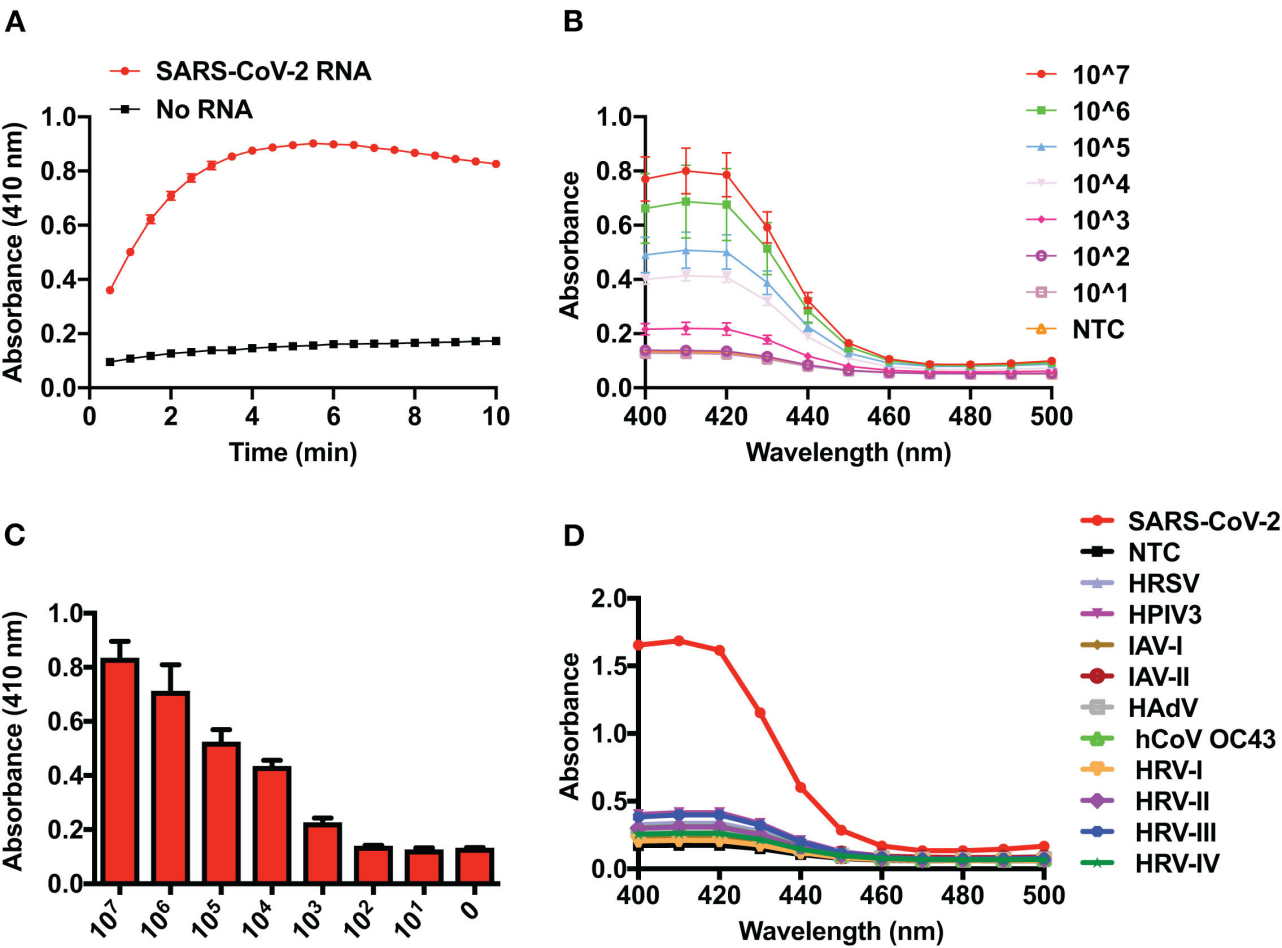
Time-dependent kinetics, limit of detection, and selectivity of the DNAzyme sensor. **(A)** Kinetics of 2,2′-azino-bis(3-ethylbenzothiazoline-6-sulfonate) oxidation by the DNAzyme sensor in the presence of SARS-CoV-2 RNA. **(B)** UV–visible spectra showing the analytical sensitivity (limit of detection) of the DNAzyme sensor. **(C)** Visual detection and its corresponding bar graph of analytical sensitivity (limit of detection) of the DNAzyme sensor and **(D)** DNAzyme sensor response in the presence of RNA of various respiratory viruses. HRSV, human respiratory syncytial virus; HPIV, human parainfluenza virus; IAV, influenza A virus; HAdV, human adenovirus; HCoV-OC43, human coronavirus-OC43; HRV, human rhinovirus.

The time taken by a sensor to detect its cognate target is a very important parameter for efficient diagnosis. Therefore, we monitored the kinetics of DNAzyme activity by observing the DNAzyme-dependent oxidation of ABTS in the presence or absence of SARS-CoV-2 RNA (Figure 2A). We observed a striking difference in DNAzyme activity in the presence or absence of SARS-CoV-2 RNA within 1 min of starting the reaction, which demonstrates the rapid activation of the DNAzyme activity when SARS-CoV-2 RNA is present. However, this difference reached its maximum limit at ~5 min, after which the signal intensity attained saturation (Figure 2A). Therefore, for all subsequent experiments, a reaction time of 5 min was chosen.

### Determination of LOD and Assessment of Cross-Reactivity

The LOD of the system is defined as the lowest amount/number of analytes/molecules detected by the sensor/diagnostic assay. Thus, to define the LOD of the DNAzyme-based sensor, we monitored the sensor response in the presence of a range (10^1^–10^7^ copies) of *in vitro* transcripts of the SARS-CoV-2 N gene. Figure 2 clearly demonstrated that as low as 1,000 copies of viral RNA can be visually detected by the DNAzyme-based sensor (Figures 2B,C). Furthermore, a concentration-dependent response of the sensor was observed by UV–visible spectra at 410 nm (Figure 2B). This detection limit is comparable to that of electrophoretogram generated on the QIAxcel platform (Supplementary Figure 2). Finally, we have also assessed the cross-reactivity of the DNAzyme with other viral RNAs (parainfluenza, influenza, adenovirus, rhinovirus, corona virus OC43, and respiratory syncytial virus). The DNAzyme sensor did not show any significant cross-reactivity with other respiratory viruses (Figure 2D). This highly selective nature of the DNAzyme sensor lies in the design of primers targeting the region of the “N” gene, which is not overlapping in the genome of other viruses.

### Clinical Evaluation

**Figure 3:**
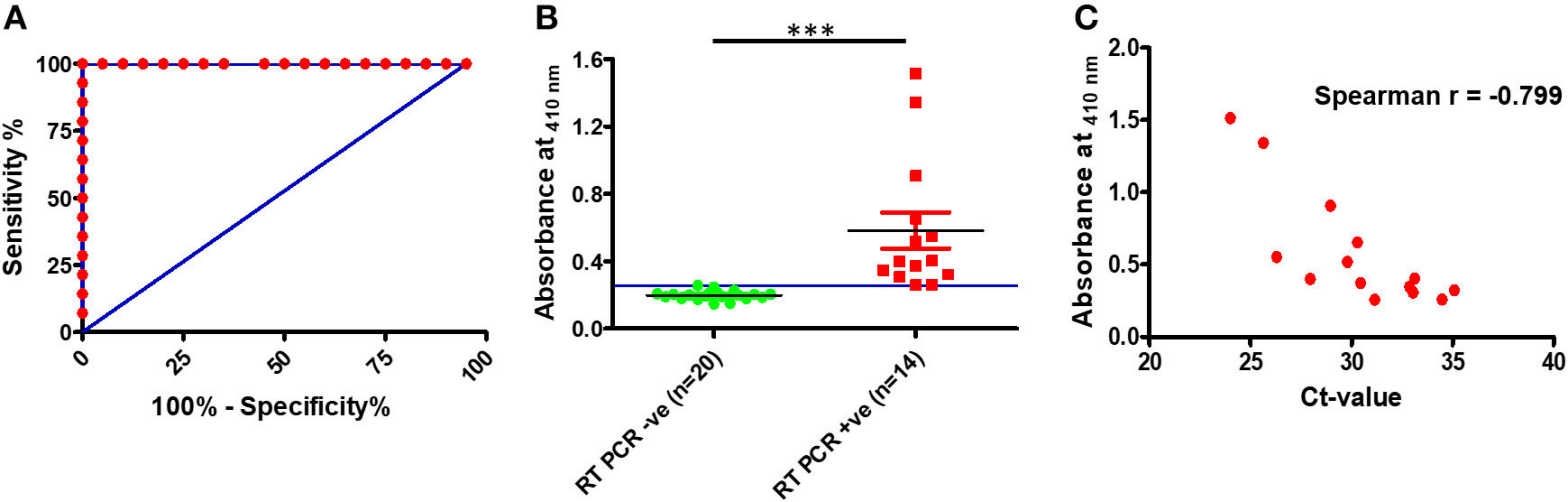
Detection of SARS-CoV-2 RNA in clinical specimens (nasopharyngeal swabs). **(A)** Receiver operating characteristic curve for the DNAzyme sensor. **(B)** Scatter plot representing SARS-CoV-2 RNA detection in nasopharyngeal swabs by the DNAzyme sensor and **(C)** correlation plot of ct values of real-time PCR and absorbance of the DNAzyme sensor. ***P < 0.001.

To assess the clinical utility of the developed assay, the performance of the RT-PCR–DNAzyme sensor was evaluated with nasopharyngeal swab specimens (*n* = 34) obtained from suspected COVID-19 patients. In addition to the RT-PCR–DNAzyme sensor, we have also evaluated the same set of NP swabs on a real-time PCR system so as to compare the performance of these two approaches (Supplementary Table 1). To determine the diagnostic sensitivity and specificity of both assays, an ROC curve was constructed (Figure 3A), and a cutoff (0.2572) line was drawn to achieve 100% specificity. It is evident from Figure 3B that the DNAzyme sensor was able to detect the presence of SARS-CoV-2 infection (viral RNA) with 100% sensitivity, and the performance of both assays was comparable in terms of sensitivity and specificity. We have also tried to establish a correlation between the threshold cycle (Ct) value of real-time PCR and the absorbance (optical density at 410 nm) of the DNAzyme-based sensor. A good negative correlation (Spearman *r* ~ −0.799) was observed (Figure 3C). Furthermore, the PPV and the NPV of the DNAzyme sensor were estimated. As expected, both the PPV and the NPV values of the DNAzyme sensor were comparable to that of the RT-PCR-based assay (Table 1). Interestingly, the DNAzyme sensor was able to detect viral RNA with Ct values as low as 35, indicating the clinical utility of our assay in COVID-19 diagnosis.

**Table 1:** Sensitivity, specificity, and negative prediction value (NPV) and positive prediction value (PPV) of the test.

**Assay**	**Sample number**	**Sensitivity (%)**	**Specificity (%)**	**PPV (%)**	**NPV (%)**
Real-time PCR	34	100	100	100	100
DNAzyme-based assay	34	100	100	100	100

## Discussion

The current diagnostic modalities for SARS-CoV-2 detection primarily rely upon molecular assays that are utilizing expensive fluorophore-labeled probes (Ravi et al., 2020). These probes that are used in commercial assays target various regions of the SARS-CoV-2 genome, including but not limited to nucleocapsid (N), spike (S) protein, RNA-dependent RNA polymerase (RdRP), or envelope (E) genes, and the ORF1b or ORF8 regions (Younes et al., 2020). In the present study, we have targeted the N gene as it is evident from the literature that, during infection of coronaviruses, the RNA encoded by N gene is in abundant quantity during early infection (Hui et al., 2004).

Unfortunately, the contemporary molecular detection assays targeting the N gene utilized fluorophore-based probes, which require detection by a real-time PCR machine. Both the probes and the real-time PCR machine are unaffordable and not widely available in many of the LMICs. Although a PCR-based approach which depends on DNA-binding dyes such as SYBR Green I for detection or visualization is a more affordable option, an inherent limitation of this assay is that an agarose gel electrophoresis setup is required for resolving and visualizing the amplicon. Casting and running of an agarose gel for a large number of samples is a tedious process. Moreover, an expensive gel documentation system or UV-transilluminator is required to visualize the amplification of viral nucleic acid. To address these existing diagnostic gaps for SARS-CoV-2, we have developed a simple visual detection assay for SARS-CoV-2 RNA using a conventional PCR. Considering the current pandemic situation and patient load, a method compatible with the conventional PCR machine with a visual read-out can be a game changer as a large number of conventional PCR machines are available across the globe. In the current study, we have developed a RT-PCR–DNAzyme sensor compatible with conventional PCR machines and demonstrated the high specificity and sensitivity of the assay using clinical samples and comparing with the gold standard, the fluorescence probe-based real-time PCR, but with the simplicity of a visual read-out. As the current assay is utilizing unlabeled primers and a DNAzyme system, it can easily be performed in a diagnostic lab having basic molecular biology facility (conventional PCR, etc.).

Previous reports have employed a similar strategy to detect both human and plant viruses (Yang et al., 2017; Kim et al., 2018; Wang et al., 2019), and a recent report has suggested the potential use of DNAzyme sensors for SARS-CoV-2 detection (Xi et al., 2020). We here demonstrate the utility of this approach for SARS-CoV-2. DNAzyme are relatively inexpensive, and their high stability makes them an attractive element for various applications (Zhou et al., 2017). We herein utilized the principle of PCR-induced generation of functional DNAzyme to detect SARS-CoV-2 RNA in NP swab samples of COVID-19 patients. The element of innovation in the current study lies in the design of the primers targeting the N gene. The developed sensor was able to detect and discriminate SARS-CoV-2 viral RNA from the RNA of other viruses. Furthermore, the developed assay was able to detect samples with Ct values as low as 35. And its clinical sensitivity and specificity in NP swabs were comparable to that of real-time PCR-based assay currently used for COVID-19 diagnosis. The good inverse correlation of the DNAzyme-based sensor with that of the Ct value of real-time PCR-based assay represents the robustness of the developed assay. Overall, the performance of the DNAzyme-based sensor is in concordance with previously published reports where DNAzyme was used for biosensing applications (Cheglakov et al., 2006; Liu et al., 2018). Taken together, the developed RT-PCR–DNAzyme sensor clearly evinced the translational potential of the assay to be used for SARS-CoV-2 testing in LMICs. In addition to the conventional gold standard of detection, an array of new approaches based on novel technology, such as a CRISPR-Cas-based detection methods, loop-mediated isothermal amplification, and smartphone-assisted electrochemical label-free sensors (Chandra, 2020; Cui and Zhou, 2020), is necessary to both bring down the cost of assays and ramp up the speed and scale of testing to flatten the COVID-19 curve. As per our cost estimates, the cost of performing a DNAzyme-based assay developed by us is around $4 as compared to $14 for a gold-standard real-time PCR assay.

We have shown that the colorimetric assay developed by our group can detect up to 1,000 copies of viral RNA (as measured by the N gene copy numbers), suggesting that the assay may miss some of the patients who have very low viral copy numbers. However, reducing disease transmission requires detecting super spreaders with high viral load in the respiratory tract (Stein, 2011). We believe that this limitation of our assay may practically not affect the purpose of COVID-19 testing at the community level. The performance of the developed assay was assessed on 34 clinical samples, which we acknowledge is a limitation. A much larger laboratory investigation on a large number of clinical samples derived from different geographical regions and ethnicity will pave the way for translating the developed sensor into a commercially viable diagnostic for the accurate detection of SARS-CoV-2.

## Data Availability Statement

The original contributions presented in the study are included in the article/Supplementary Materials, further inquiries can be directed to the corresponding authors.

## Ethics Statement

The study was approved by the ethics committee of THSTI THS 1.8.1/(91) dated 13 April 2020. RNA samples from other respiratory virus infections were used from a study described previously (Kumar et al., 2017), which was a cohort of newborns who were followed for the first year of life, for each episode of acute respiratory infection. Nasopharyngeal aspirates were collected to identify the causative respiratory viruses using multiplex real-time PCR assay. This study was approved by the institutional ethics committees of THSTI, Faridabad [THS/1.8.1(12)], and AIIMS New Delhi (IEC/P-13512012 and RP-O6/2O12).

## Author Contributions

AA, SD, and PS conducted the investigation, validation, visualization, and formal analysis. RL and SK contributed to the resources, writing, review, and editing of the manuscript, and formal analysis. TS and GM contributed to the conceptualization, methodology, validation, formal analysis, writing of the original draft, review and editing, and supervision. All authors contributed to the article and approved the submitted version.

## Conflict of Interest

The authors declare that the research was conducted in the absence of any commercial or financial relationships that could be construed as a potential conflict of interest.
