# Supplementary material for: Visual Detection of SARS-CoV-2 RNA by Conventional PCR-Induced Generation of DNAzyme Sensor
Source: Front Mol Biosci. 2020 Dec 23;7:586254. doi: 10.3389/fmolb.2020.586254 (PMC7793695; doi:10.3389/fmolb.2020.586254)
Supplement: Supplementary file 1 [file Data_Sheet_1.pdf]

Supplementary Information

Supplementary Table S1: Diagnostic performance of RT-PCR-DNAzyme sensor in clinical specimens (nasopharyngeal swabs n=34)

S.No.	Ct-value of Real time PCR	DNAzyme O.D. at 410 nm	Visual detection
1.	24.01	1.5114	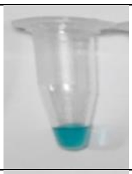
2.	25.64	1.3428	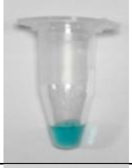
3.	26.29	0.552	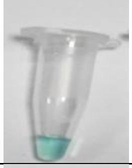
4.	27.96	0.4009	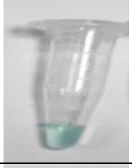
5.	28.96	0.9073	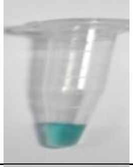
6.	29.80	0.519	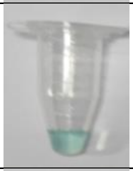
7.	30.3	0.6544	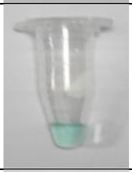
8.	30.45	0.3722	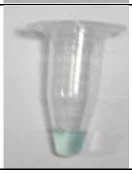

9.	31.15	0.2583	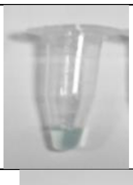
10.	32.87	0.3475	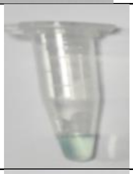
11.	33.05	0.3068	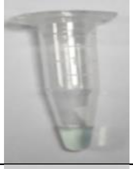
12.	33.13	0.4039	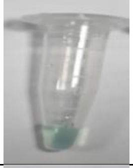
13.	35.1	0.3218	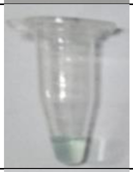
14.	36.27	0.2588	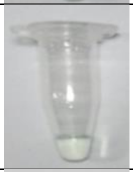
15.	NA	0.2047	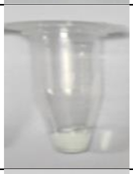
16.	NA	0.2078	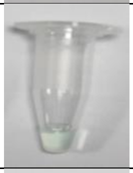
17.	NA	0.2029	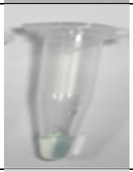
18.	NA	0.1909	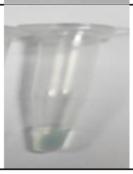

19.	NA	0.2561	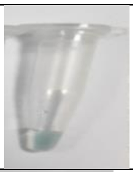
20.	NA	0.2114	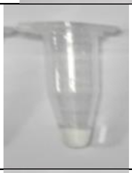
21.	NA	0.1897	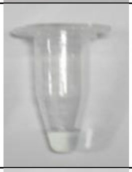
22.	NA	0.1476	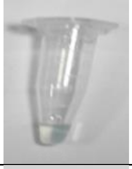
23.	NA	0.1996	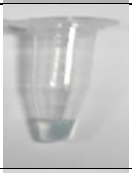
24.	NA	0.1799	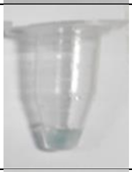
25.	NA	0.1866	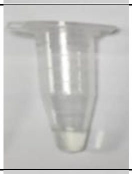
26.	NA	0.1810	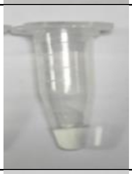
27.	NA	0.1952	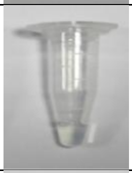
28.	NA	0.2272	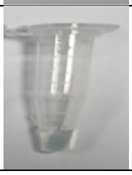

29.	NA	0.2010	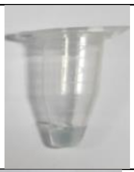
30.	NA	0.2029	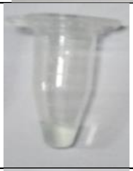
31.	NA	0.1757	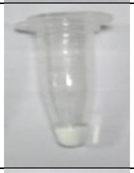
32.	NA	0.2144	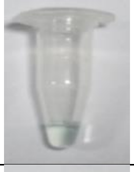
33.	NA	0.1501	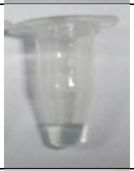
34.	NA	0.2472	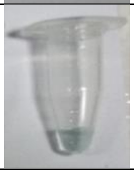

Supplementary Figure S1

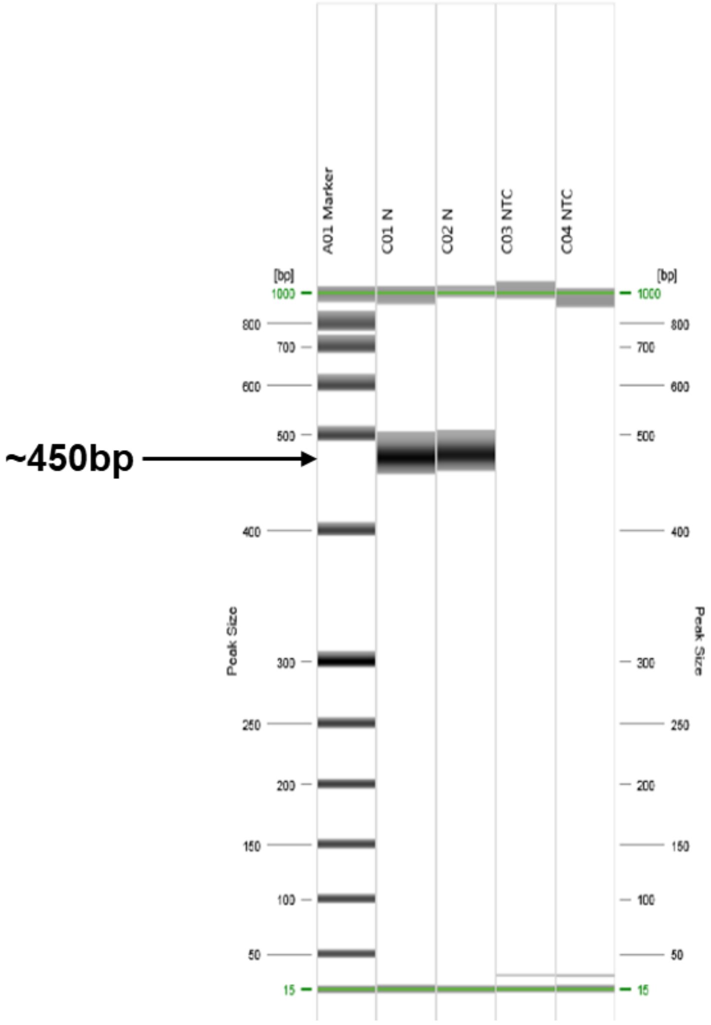

Figure S1: QIAxcel electrophoretogram representation of amplification of SARS-CoV-2 RNA through RT-PCR. Lane C01 and C02 represent N gene specific amplification corresponding to ~450 bp. NTC represent no template control where no RNA was added.

Supplementary Figure S2

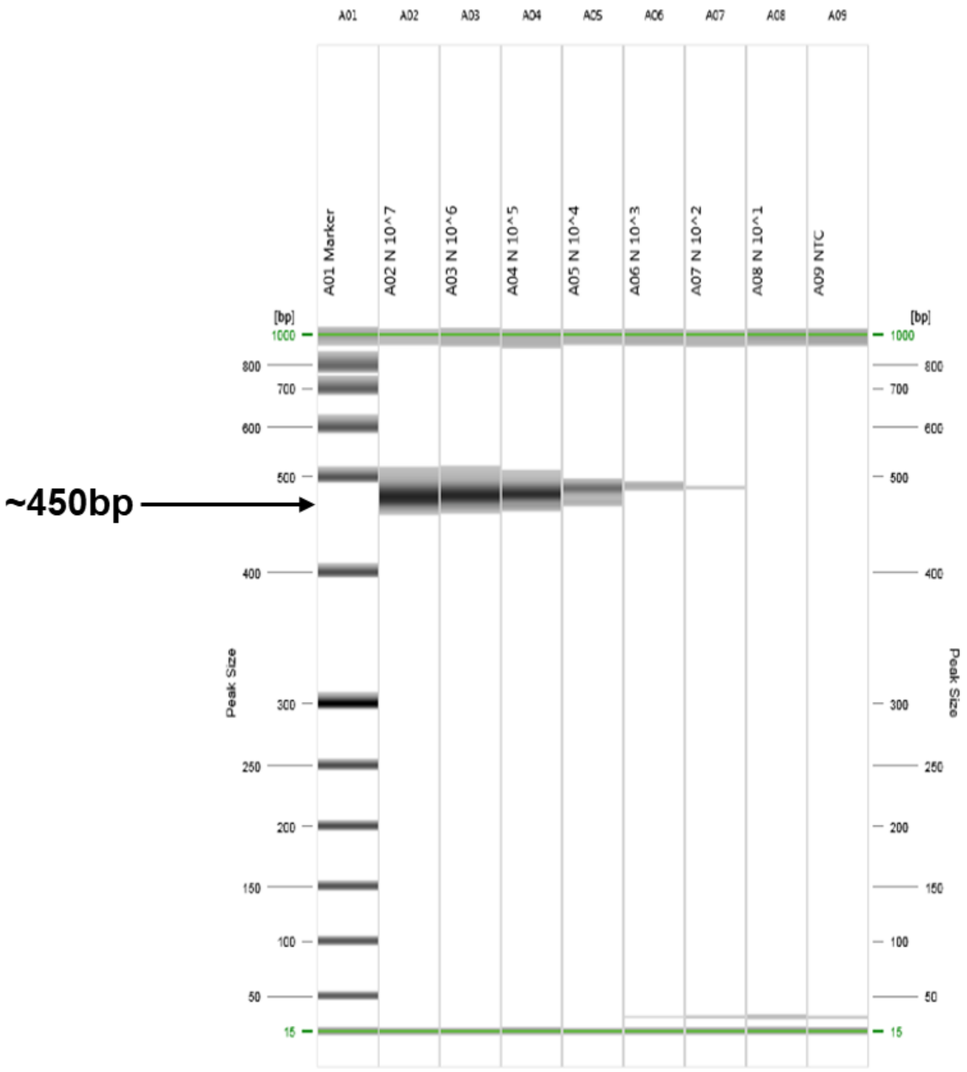

Figure S2: Analytical sensitivity, QIAxcel electrophoretogram representing amplification with respect to *in vitro* transcript amount. Lane A01 represent ladder. Lane A02-A08 represent analytical sensitivity in decreasing order (from 10^7 to 10^1 copies). NTC represent no-template control where no RNA was added.
